# Orientation Dependent MR Signal Decay Differentiates between People with MS, Their Asymptomatic Siblings and Unrelated Healthy Controls

**DOI:** 10.1371/journal.pone.0140956

**Published:** 2015-10-21

**Authors:** Enedino Hernández-Torres, Vanessa Wiggermann, Simon Hametner, Tobias R. Baumeister, A. Dessa Sadovnick, Yinshan Zhao, Lindsay Machan, David K. B. Li, Anthony Traboulsee, Alexander Rauscher

**Affiliations:** 1 UBC MRI Research Centre, University of British Columbia, Vancouver, Canada; 2 Department of Pediatrics, University of British Columbia, Vancouver, Canada; 3 Department of Physics and Astronomy, University of British Columbia, Vancouver, Canada; 4 Department of Neuroimmunology, Center for Brain Research, Medical University of Vienna, Vienna, Austria; 5 Department of Electrical and Computer Engineering, University of British Columbia, Vancouver, Canada; 6 Pacific Parkinson's Research Centre, University of British Columbia, Vancouver, Canada; 7 Department of Medicine (Neurology), University of British Columbia, Vancouver, Canada; 8 Centre for Brain Health, University of British Columbia, Vancouver, Canada; 9 Department of Medical Genetics, University of British Columbia, Vancouver, Canada; 10 Department of Radiology, University of British Columbia, Vancouver, Canada; 11 Child and Family Research Institute, University of British Columbia, Vancouver, Canada; University of Düsseldorf, GERMANY

## Abstract

R2* relaxometry of the brain is a quantitative magnetic resonance technique which is influenced by iron and myelin content across different brain regions. Multiple sclerosis (MS) is a common inflammatory, demyelinating disease affecting both white and grey matter regions of the CNS. Using R2*, increased iron deposition has been described in deep gray matter of MS patients. Iron accumulation might promote oxidative stress in the brain, which can lead to cell death and neurodegeneration. However, recent histological work indicates that iron may be reduced within the normal appearing white matter (WM) in MS. In the present study we analyzed the R2* signal across the white matter in 39 patients with MS, 31 asymptomatic age matched siblings of patients and 30 age-matched controls. The measurement of R2* in white matter is affected by the signal's dependency on white matter fibre orientation with respect to the main magnetic field which can be accounted using diffusion tensor imaging. We observed a clear separation of the three study groups in R2*. The values in the MS group were significantly lower compared to the siblings and controls, while the siblings group presented with significantly higher R2* values than both unrelated healthy controls and patients. Furthermore, we found significantly decreased normal-appearing white matter R2* values in patients with more severe disease course. Angle resolved analysis of R2* improves the sensitivity for detecting subtle differences in WM R2* compared to standard histogram based analyses. Our findings suggest that the decreased R2* values in MS are due to diffuse tissue damage and decreased myelin in the normal appearing and diffusely abnormal WM. The increased R2* in unaffected siblings may identify a predisposition to increased iron and the potential for oxidative stress as a risk factor for developing MS.

## Introduction

Multiple sclerosis (MS) is a chronic inflammatory disease of the central nervous system, affecting white and grey matter in the brain, spinal cord and optic nerves. While the cause of MS is unknown, both genetic and environmental factors and interactions of these are thought to play a role. Within families, the risk for biological relatives of patients with MS to develop the disease is associated with the degree of DNA sharing [1–3] with risks for half-siblings, full siblings, dizygotic twins and monozygotic twins being 1.89%, 3.11%, 5.4%, and 25.4% [4, 5] compared to 0.13% in the general population [6]. However, the roles of non-genetic gestational and neonatal factors due to a shared uterine and postnatal environment must also be considered, given the higher MS risk of dizygotic twins versus full siblings [4].

Focal lesions in the white matter (WM) of the central nervous system (CNS) are the pathological and radiological hallmark of MS. These lesions are characterized by a variable degree of episodic inflammation, demyelination of axons, gliosis, and axonal injury [7]. New and active focal WM lesions are commonly seen in patients having a relapsing-remitting disease course (RRMS), while progressive stages of MS show more pronounced cortical demyelination and diffuse injury of the normal appearing WM (NAWM) [8]. However, all stages of MS have evidence of diffuse tissue damage beyond the typical focal lesions.

Recently, studies have proposed R2* relaxation as a sensitive measure of tissue damage in focal MS lesions [9], and as a marker for iron accumulation in the deep gray matter [10–12]. Iron accumulation in the brain is believed to promote oxidative damage and thus is a topic of interest with regard to many neurodegenerative diseases [13]. While iron deposition within the deep gray matter of patients with MS has been extensively studied [11, 12], little is known about iron dynamics within the WM. Recent histological and MRI studies suggest that the iron content in the non-lesional WM in patients with MS might be reduced compared to age-matched controls [13,14]. R2* histograms across the whole WM of patients with MS are shifted towards lower R2* values compared to histograms of healthy controls (HC) [9] due to the reduced R2* in regions of tissue damage. As well, the signal's dependency on tissue orientation relative to the main magnetic field [15, 16], contributes to further broadening of the histograms. Therefore, the averages calculated from the R2* distribution across the whole WM without correction for orientation may affect the accuracy and precision of the values.

Here, we investigated R2* relaxation rates in the non-lesional WM of patients with MS, their unaffected full siblings with no evidence of neurological damage and unrelated healthy controls (HC), accounting for tissue orientation using diffusion tensor imaging (DTI). We hypothesize that resolving the signal by its angle dependency (orientation) allows a more sensitive estimate of WM changes between these different groups than comparing whole brain histograms. We further believe that such an R2* analysis, encompassing the whole non-lesional WM of the brain, is well-suited to uncover diffuse MRI changes possibly due to changes in water, myelin or iron contents in the WM of both MS patients and their siblings, when compared to unrelated HC. The unaffected siblings cohort allows us to assess potential tissue abnormalities that could be associated with familial risk of MS.

## Methods

### Standard protocol approval, registrations and patient consents

The Clinical Research Ethics Board of the University of British Columbia approved the study protocol and all subjects gave written informed consent in accord with the Declaration of Helsinki.

### Subjects

Three groups of individuals were assessed (i) patients with a diagnosis of MS, (ii) full siblings of patients with MS who do not themselves show any clinical signs or symptoms of MS and (iii) healthy volunteers.

### Data acquisition

All participants were scanned on a 3T system (Philips Achieva) using an 8-channel SENSE head coil. Data for R2* mapping were collected using a three dimensional gradient-echo sequence with five echoes (TR = 28 ms, TE1 = 5 ms, echo spacing = 5 ms, α = 17°, field of view = 230 x 165 x 110 mm^3^, acquired voxel size = 0.9 x 1 x 1.6 mm^3^, reconstructed voxel size = 0.8 x 0.8 x 0.8 mm^3^). DTI data were acquired using a spin echo planar sequence with b0 = 0, b1 = 1000, 16 directions, TR/TE = 7465/75 ms, FOV = 212 x 212 mm^2^, 60 slices of 2.2 mm thickness, in-plane resolution = 2.2 x 2.2 mm^2^. Three-dimensional T1 and T2 weighted scans were collected for image registration purposes and WM segmentation with the following parameters: 3D T1-weighted sequence: voxel size = 1.0 x 1.0 x 1.6 mm^3^, reconstructed to 0.8 x 0.8 x 0.8 mm^3^, FOV = 256 x 256 x 160 mm^3^, TR/TE = 7.6/3.7 ms. 3D T2-weighted scan: voxel size = 1.0 x 1.0 x 1.6 mm^3^, reconstructed to 0.8 x 0.8 x 0.8 mm^3^, FOV = 256 x 256 x 160 mm^3^, TR/TE = 2500/363 ms.

### Data processing and analysis

**Fig 1:**
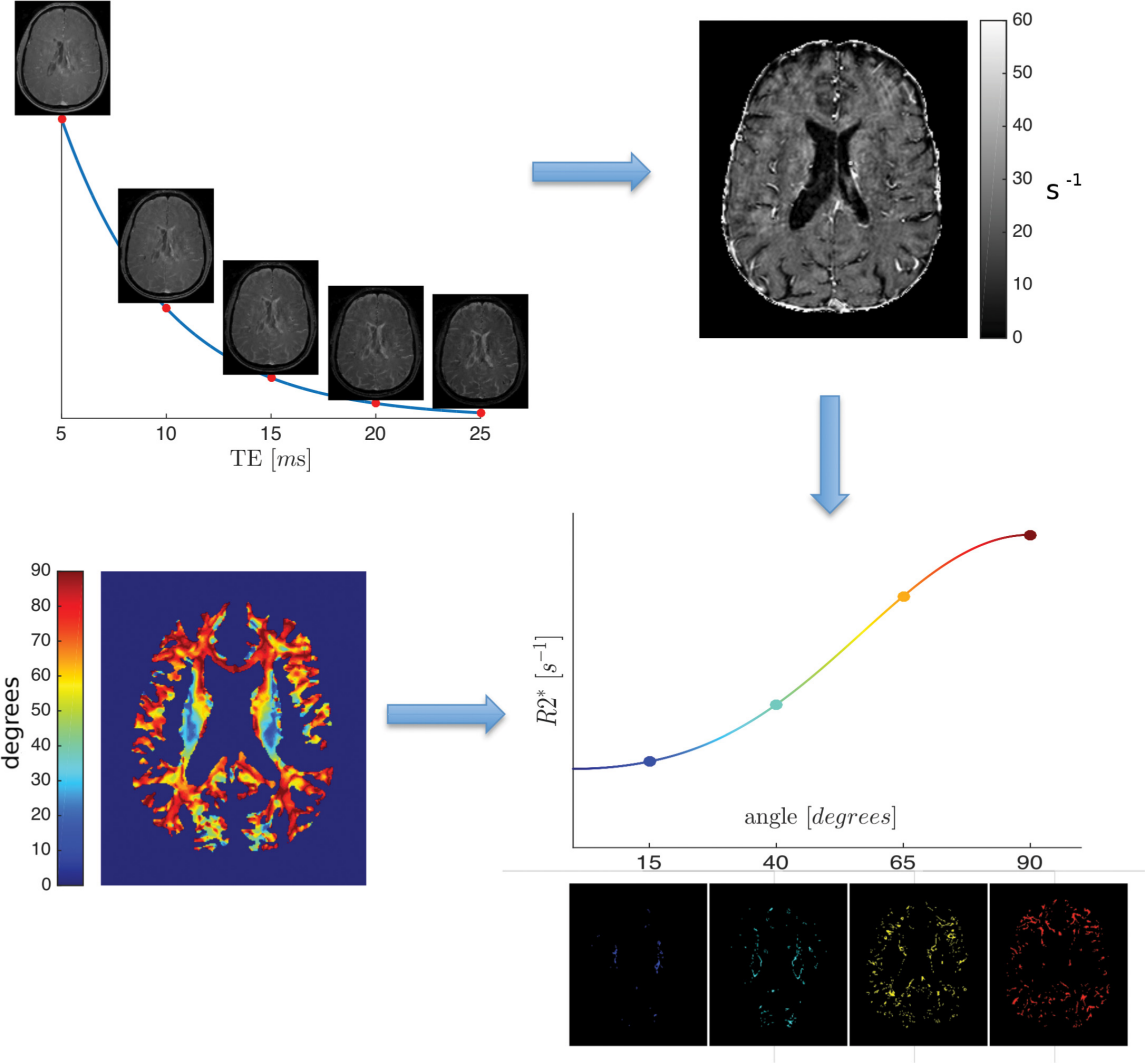
Data processing workflow. From multi echo gradient echo data, an R2* map is computed. The angle between the largest eigenvector of the diffusion tensor and B_0_ is computed in each voxel. The R2* values from each orientation interval are pooled together, averaged and then plotted against the corresponding orientation. The four exemplary images below the R2* curve represent four angle intervals (10–15, 35–40, 60–65, and 85–90 degrees, respectively) and show which voxels contribute to the R2* average. Their color corresponds to the color of the angle map.

R2* maps were calculated from the multi gradient-echo scans by fitting a mono-exponential function to the signal decay in each voxel. DTI data were processed using FSL [17]. Eddy current and head motion correction of DTI data were performed via a linear, affine registration (FLIRT) and non-brain voxels were removed using FSL's brain extraction tool (BET). Eigenvalues and their corresponding eigenvectors were calculated using FSL's DTIFIT. T1, T2 and DTI data were linearly registered to the R2* maps using FLIRT. The combination T1T2 = (T1—T2) / (T1 + T2) enhances contrast between WM, GM and subcortical structures [18], and was used for tissue segmentation with FSL’s FAST [18]. The obtained WM masks were eroded using a 5-voxel kernel. Additional manual correction was performed to remove non-WM voxels. Focal lesions were manually defined based on both the T1 weighted images and the T1T2 images to increase confidence in lesion identification. Analysis of R2* values was performed for WM after the removal of focal lesions from the WM mask. Conventional histograms of R2* values within the non-lesional WM were computed. Maps of the angle between the main magnetic field and the principal diffusion direction of every voxel as a measure of local fibre orientation were computed spanning angles between 0 and 90 degrees [15]. We pooled all voxels with orientations within 5 degree intervals and used them to calculate the average R2* relaxation as a function of the fibre orientation (Fig 1). Furthermore, the behaviour of those curves was investigated using the model presented in [19] which accounts for the orientation dependency of WM structures as well as magnetic susceptibility anisotropy in WM:

$$R_{2}^{*}=c_{0}-c_{1}.\text{cos}\left(2\theta \right)+c_{2}.\text{cos}\left(4\theta \right)$$ Eq. 1

According to Eq 1, R2* is governed by an orientation (θ) independent component c_0_ and two additional terms representing fibre orientation dependent isotropic and anisotropic contributions to the R2* signal. The isotropic component c_1_ cos(2θ) stems from the macroscopic cylindrical geometry of WM fibres while the anisotropic component c_2_ cos(4θ) arises from the microscopic myelin structure. The coefficients c_1_ and c_2_ modulate the angular dependency of both the isotropic and anisotropic component, respectively. Fitting was performed using a least squares approach. Further, we compared the R2* angle-resolved behaviour with clinical variables, such as the disease stage, e.g. RRMS, secondary/primary progressive (SPMS/PPMS), as well as with their expanded disability status scale (EDSS) and estimated disease duration (DD). To assess the influence of the disease progression on the observed R2* values, we determined the progression index (PI = EDSS/DD) for each patient [20].

### Statistical Analysis

Statistical analysis was performed using R (R Agricolae package [21]). For simultaneous comparison of the 3 groups (MS, siblings, HC), a Kruskal-Wallis test was performed. Further pairwise comparisons were carried out using a Kruskal-Wallis test with Holms adjustment for multiple comparisons. We assessed the individual dependency of the coefficients c_0_, c_1_, and c_2_ on age, DD and EDSS using Spearman correlations. Furthermore, we investigated the influence of age and sex on the observed R2* curves.

## Results

For this study we recruited 39 patients with MS (32 RRMS, 3 PPMS, and 4 SPMS, Table 1). Thirty-one full siblings of the patients with MS were scanned (2 monozygotic twins, 8 dizygotic twin, 21 singleton siblings), as well as 30 unrelated HC.

**Table 1:** Demographics of subjects. *EDSS: Expanded disability status scale [22].

	Patients	Non-MS full Siblings	Healthy Controls	*p*-value
**N (female/male)**	39 (35/4)	31 (20/11)	30 (25/5)	0.03
**Age (years)**	[32–69] 49.7±10.1	[30–67] 50.6±11.0	[30–67] 50.6±11.3	0.88
**Age at disease onset (years)**	[17–56] 32.4±8.0	NA	NA	NA
**Disease Duration (DD, years)**	[3–41] 17.2±9.3	NA	NA	NA
EDSS*	[0–6.5] median 2.5	NA	NA	NA

Three siblings and three unrelated controls had WM abnormalities on T2-weighted scans but were clinically asymptomatic. These 6 subjects were excluded from further analysis. 27 of the remaining 28 siblings were siblings of MS patients in our cohort. Two of the siblings were related to the same patient, one sibling was related to two MS patients.

**Fig 2:**
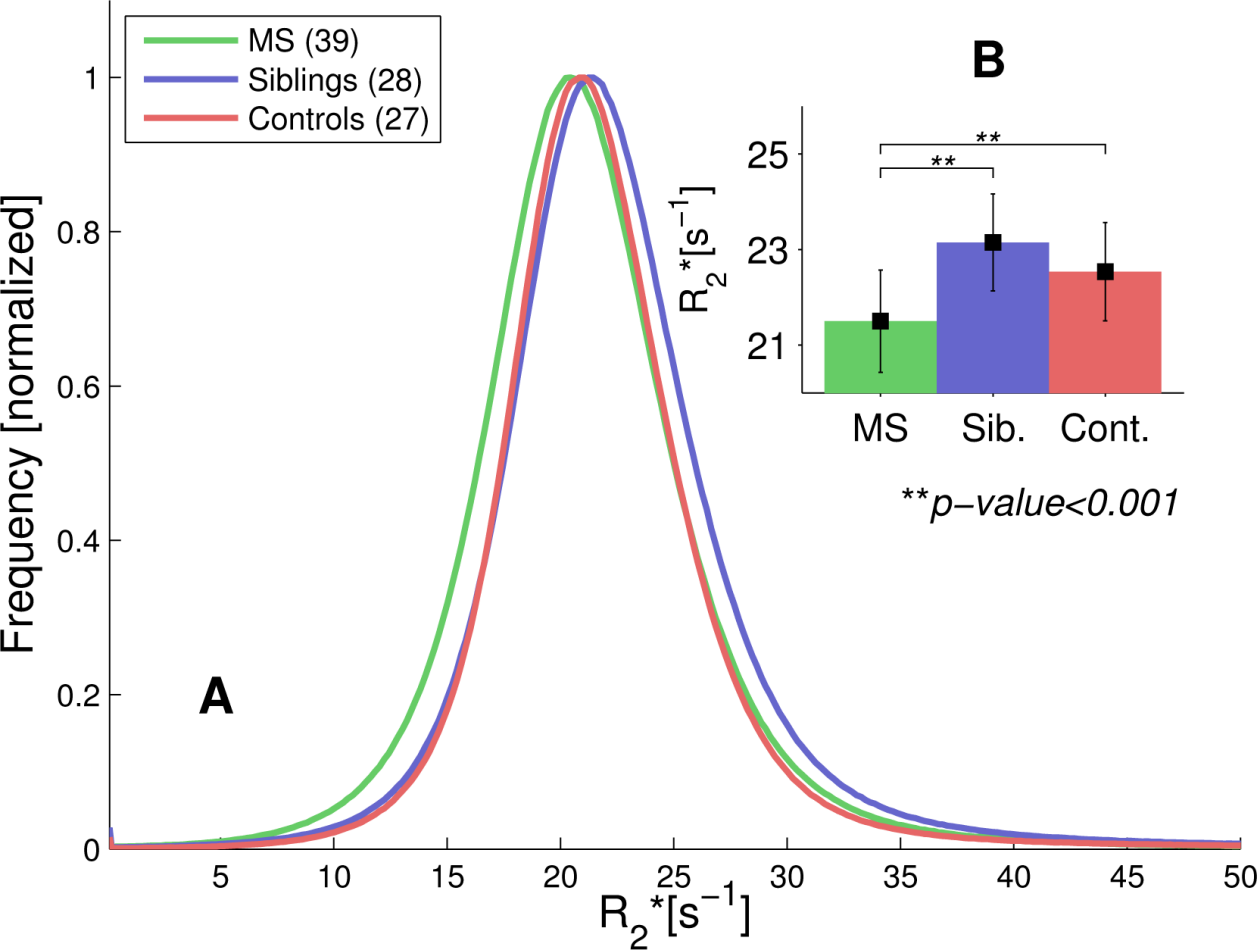
R2* values within the whole WM. (A) Normalized R2* histograms across the entire non-lesional WM in all three cohorts. The histograms for controls and siblings have a large overlap, the MS group's histogram, on the other hand, is shifted towards lower R2* values. (B) The insert shows average R2* values and standard deviations of the respective groups. The R2* average was significantly reduced in the MS cohort (p < 0.001), but no difference was observed between controls and siblings.

The normalized R2* histograms of the non-lesional WM are shown in Fig 2A. The peak of the R2* histogram of the MS group is shifted towards lower R2* values compared to the sibling (*p* < 0.001) and HC (*p* < 0.05) groups, while the histograms obtained for siblings and unrelated controls overlap strongly (*p* > 0.05). The average and standard deviation of the WM R2* histograms are 21.50 ± 1.07s^-1^ in MS patients, 23.15 ± 1.01 s^-1^ in the siblings and 22.54 ± 1.03 s^-1^ in unrelated HC, presented in the inset bar plot (Fig 2B).

**Fig 3:**
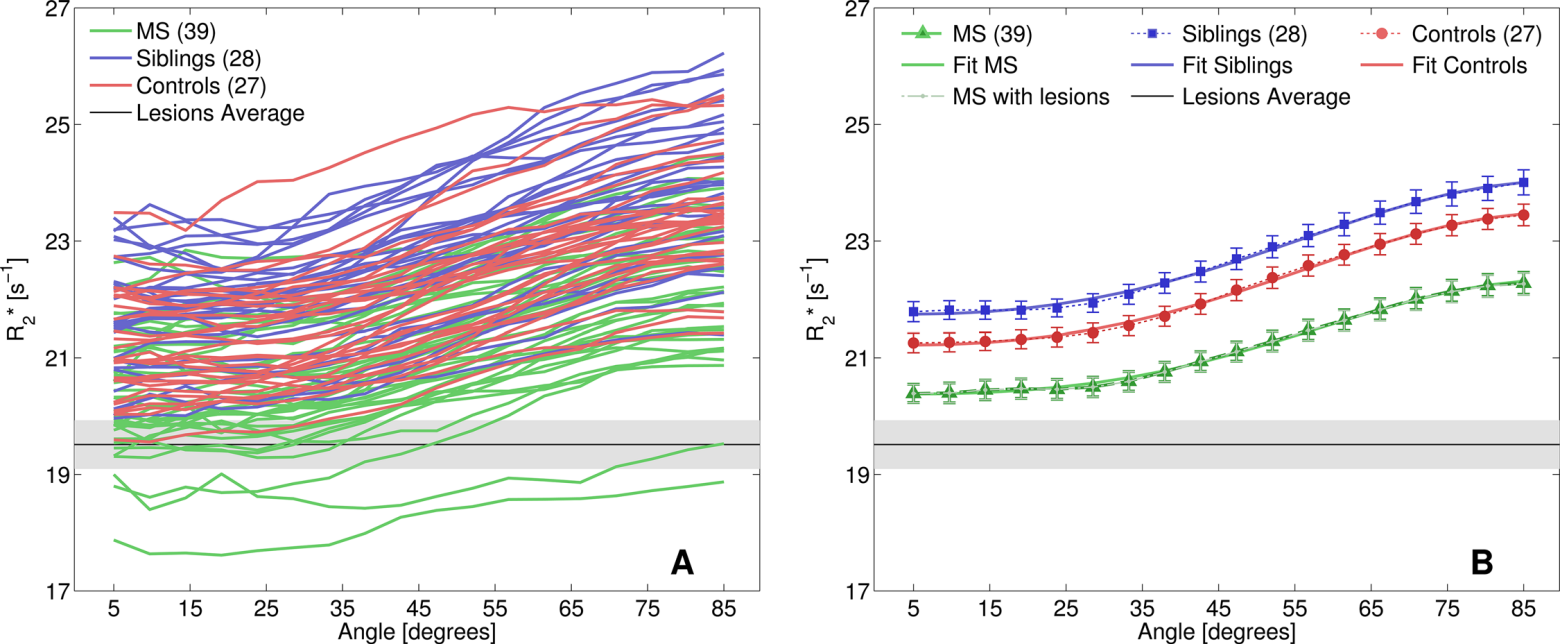
R2* as a function of the fibre orientation. (A) R2* values in the WM as a function of fibre orientation relative to B_0_ for each subject in the three cohorts. The shaded area in (A) and (B) indicates mean (black line) and standard error (gray area) of R2* values in MS lesions. The R2* values are the averages of all voxels with fibre orientations within 5 degree intervals. (B) The mean R2* and standard errors for MS, siblings and controls are represented by triangles, squares, and circles, respectively. Solid lines reflect the fit to the mean of each group according to Eq 1. Dark green shows the behaviour of R2* in WM, excluding lesions, while light green depicts the R2* behaviour in all WM voxels.

All subjects showed a clear dependency of R2* on tissue orientation with respect to the main magnetic field (Fig 3A). At all angles, the patients with MS had lower R2* values than siblings and HC. Conversely, the sibling group, exhibited elevated R2* values compared to HC and patients with MS.

Fitting the model (Eq 1) to the R2* angle-dependent data of each individual subject yielded distributions of the three fitting coefficients c_0_, c_1_, and c_2_ (Table 2). The average for the orientation independent coefficient, c_0_, was highest in the sibling group, followed by the HC and the MS group. Upon correction for multiple comparisons (three groups), differences in c_0_ remained significant between the MS group and both the healthy siblings and HC (*p* < 0.001), but the difference between the sibling and HC group did not reach significance (*p* = 0.065). Additionally, correlations of c_0_ with age showed a weak positive trend, which was significant (*p* = 0.03) only in the siblings group. In contrast, neither the isotropic parameter c_1_ nor the anisotropic fitting coefficient c_2_ showed significant differences between the groups or any correlation with age. The fitting coefficients did not correlate with EDSS (*p* = 0.1913) or DD in the MS group (*p* = 0.1361). Supplementary file S1 File contains the R2* curves for each subject as well as subject specific information, including group (control, sibling, patient), age, sex, EDSS, and disease duration.

**Table 2:** Distribution of the fitting parameters for all groups: Significant differences were found for c_0_ between MS patients and siblings and MS patients and controls while only a trend to significance was observed between siblings and controls.

	c_0_ [range] (mean±std) [s^-1^]	c_1_ [range] (mean±std) [s^-1^]	c_2_ [range] (mean±std) [s^-1^]
**Patients**	[18.25–23.04] 21.22±1.05	[0.36–1.75] 0.98±0.33	[-0.20–0.54] 0.14±0.15
**Siblings**	[20.95–24.47] 22.81±0.95	[0.35–2.30] 1.14±0.29	[-0.16–0.36] 0.12±0.12
**Controls**	[20.56–24.65] 22.22±0.97	[0.65–1.87] 1.14±0.28	[-0.30–0.54] 0.13±0.17

Fitting of the average orientation dependent R2* values across all subjects in their respective subject group resulted in three orientation dependent functions for R2* (solid lines in Fig 3B). The coefficients of determination were R^2^ = 0.997 for the MS group, R^2^ = 0.997 for the siblings and R^2^ = 0.998 for the unrelated control group. The R2*-orientation dependency in patients did not change if lesion tissue was included in the analysis (dashed light green line in Fig 3B). The fitted curves are predominantly separated by an offset defined by their respective R2* value at θ = 0. The highest R2* values in each group were observed in fibres perpendicular to the main magnetic field (B_0_), while the lowest R2* values were found in fibres parallel to B_0_. The difference between fibres parallel and perpendicular (∆R2*) was smallest in the MS group (1.89 ± 0.66 s^-1^), followed by the HC (2.21 ± 0.53 s^-1^) and siblings (2.21 ± 0.66 s^-1^).

Apart from the fitting coefficients, we can contrast the average R2* values of each angle interval between the three groups. We observed significant differences when comparing the MS population to the HC (*p* < 0.003) and the siblings group (*p* < 0.001). The differences were also significant when only the RRMS group was compared to matched HC and siblings. Controls versus siblings showed significant differences in R2* (*p* < 0.05) for most angle intervals; with angles larger than 80 degrees presenting a trend towards significance (*p <* 0.058).

**Fig 4:**
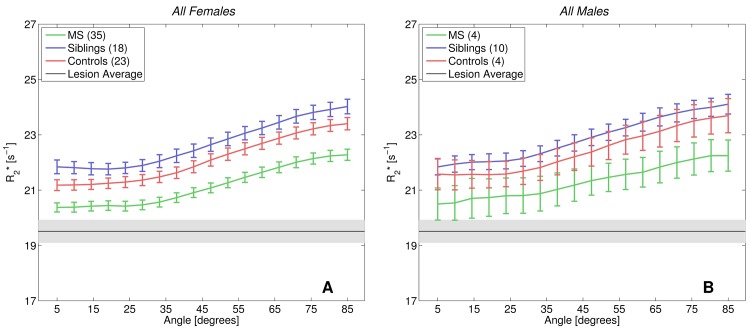
R2* values of all cohorts divided by sex. The comparison of R2* values in only female (A) and only male (B) cohorts show the same pattern of separation between the groups as observed in Fig 3B, suggesting that these differences are not driven by different numbers of males and females in the three cohorts. All female and male cohorts are closely age matched.

Fig 4 depicts the influence of sex on the orientation-dependent R2* curves in age matched groups, showing that the difference between the MS, siblings and unrelated control groups observed in Fig 2 is independent of the sex ratio in each group.

To investigate the possible relation of R2* reduction and the course of the disease, the MS group was first subdivided according to the patients’ disease stage (e. g. RRMS vs progressive MS (PMS), which is pooled secondary and primary progressive). These groups showed no significant R2* differences (*p* > 0.05, results not shown).

**Fig 5:**
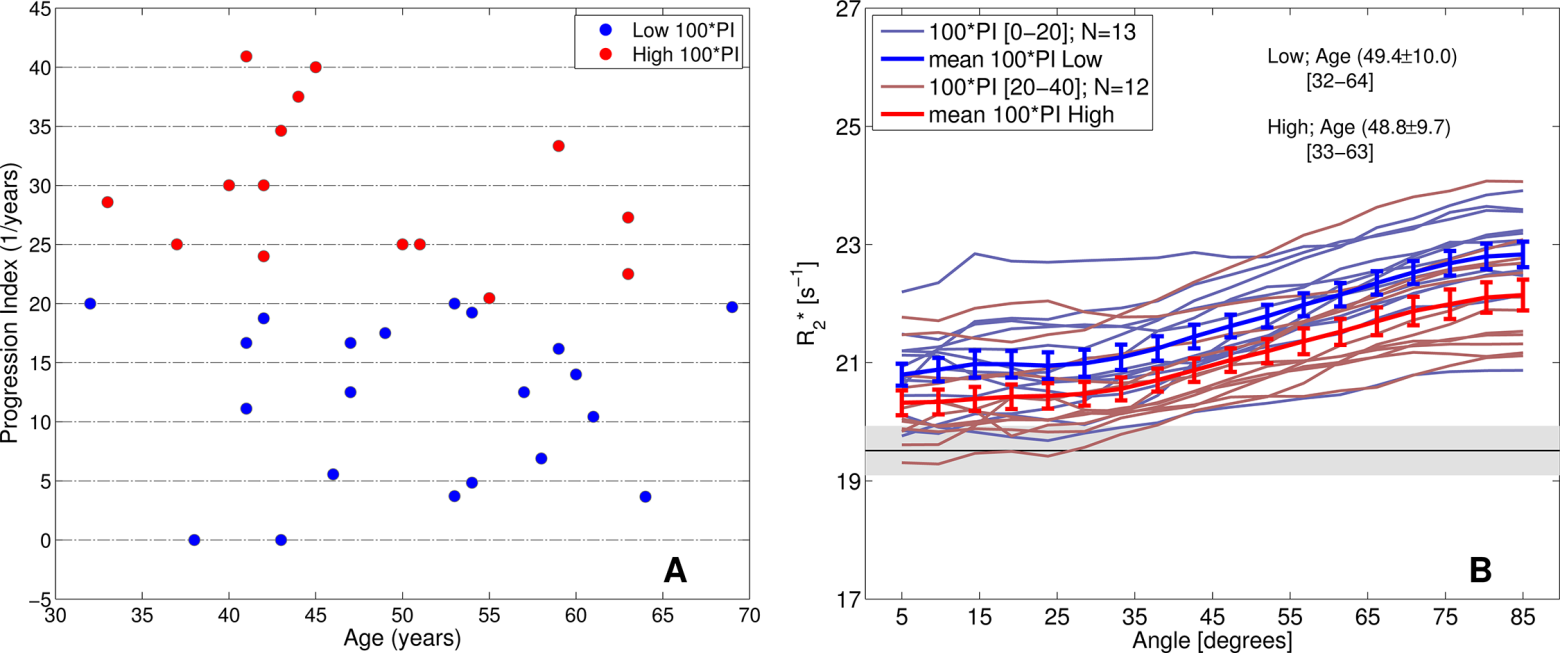
Progression index. Progression index for all participants of the MS cohort, calculated as EDSS/DD (DD = disease duration). (A) Low and high PI values were separated based on a cut-off at 100*PI = 20. (B) Age-matched patients were divided in high 100*PI (>20) and low 100*PI (<20). Note that R2* values in the low PI group are higher than in the high PI group, supporting the hypothesis of a decrease in R2* with disease progression.

For further analysis, we separated the patients group by their progression index (PI) scores in groups of 100*PI < 20 and 100*PI > 20. In contrast to EDSS and DD, the PI showed no correlation with age (Fig 5A). We found that patients with higher PI exhibited smaller R2* values, which was significant in the interval [45–90] degrees; *p* < 0.05 (Fig 5B). Therefore, an association between PI and R2* is not confounded by age, which is known to be correlated with WM iron values in HC.

## Discussion

### Angle resolved R2* analysis

Resolving the angle dependency of R2* resulted in a separation of patients with MS, siblings of patients with MS, and unrelated HC. This improved resolution is achieved through the removal of the angle dependent broadening encountered in R2* histograms. We further demonstrated that R2* at angles closer to 90 degrees in patients may be similar to R2* values at lower angles in the other two groups (Fig 3B). This effect contributes to the large overlap of the histograms (Fig 2A) and hides subtle differences, mainly between siblings and HC. The theoretical model fits the data in all three groups remarkably well. While iron is a strong modifier of R2*, it was shown previously that it is not responsible for the orientation dependency in R2*. Experiments where iron was extracted from the *corpus callosum* and *basal ganglia* reported an overall decrease of R2*, however, the angle dependency was unaffected [19]. Therefore, alterations of iron content will affect c_0_ rather than c_1_ or c_2_. Although there were no significant group differences for the fitting coefficients c_1_ and c_2_, which modulate the angle dependent cosine terms in Eq 1, the difference in R2* between parallel and perpendicular fibres (∆R2*) was smallest in the MS patients [19]. This suggests that the changes of the NAWM in our MS group are in part represented by c_1_. Axonal degeneration [23] and degradation of the myelin sheath accompanied by the accumulation of myelin debris followed by its clearance and the formation of a glial scar may explain the reduced ∆R2* in patients due to changes in the tissues architecture. This interpretation is in agreement with the reduction in magnetization transfer ratio [24], as well as reduced R2 and myelin water fraction in diffusely abnormal WM (DAWM) that correlated well with reduced staining with Luxolfast Blue, a marker for myelin phospholipids [25]. Given that a reduction in myelin in NAWM and DAWM happens early in MS [26, 27], the absent or small differences in c_1_, c_2_ and ∆R2* are surprising. In lesions these three parameters vanish, which is in agreement with the profound demyelination in this tissue. However, this observation has to be interpreted with some caution as axonal loss introduces noise in the orientation measurement. To avoid a systematic bias towards tissue with higher FA, no FA threshold was applied, which in turn may result in additional noise in the orientation measurement due to kissing or crossing fibres.

### Elevated R2* in siblings of MS patients

Siblings of people with MS have an increased lifetime risk to develop MS compared to the general population [3]. However, our sibling group is on average 50 years and therefore beyond the typical age of disease onset. Studies for singular genetic, epigenetic or transcriptomic mechanisms did not find conclusive differences between monozygotic twins discordant for MS, while such differences were found in comparison with unrelated HC [28]. Those results are strongly suggestive of genetic similarity and predisposition of siblings for developing MS. Such a predisposition for MS may very well be reflected as non-lesional changes on MRI in the siblings and may need additional environmental factors on such a shared genetic background. One may speculate that the siblings' increase in R2* is due to an increased iron content in their WM. It has been suggested that oxidative stress mediated by excess iron may play a role in the etiology of MS [29, 30], however to our knowledge there are no studies that investigate the presence of iron in early stages of MS in WM that further support this hypothesis of increased WM iron being among MS-predisposing conditions of siblings. Increased iron concentrations may be one of several components that ultimately contribute to MS but in the absence of additional components, iron by itself may not be sufficient to cause MS. Only a *post mortem* analysis of sibling brains would allow for definite conclusions on brain iron content in this group. The similar ∆R2* between siblings and controls further supports that the orientation dependent macroscopic and microscopic structural integrity is unaffected in the siblings and that an orientation independent mechanism is responsible for the overall R2* increase in siblings. Another modifier of R2* is blood oxygenation, which is influenced by cerebral blood flow and oxygen extraction. The R2* decay around blood vessels depends on the degree of oxygenation within these vessels [31]. One may speculate that the siblings group extracts more oxygen from the arterial blood than the control group, leaving the venous blood less oxygenated, which could result in an increase in c_0_. On the other hand, oxygen metabolism was found to be reduced in patients with MS, which means that their venous vasculature would carry more oxygen than that of controls, resulting in further reduction of R2* in patients [32].

A contributor to the increase of R2* observed in the siblings group could be the higher percentage of males in this group. Differences in body iron stores between males and females are best documented in the form of higher hemoglobin iron levels seen in the males due to different hematocrits. Additionally, males present higher serum ferritin levels indicative of body iron stores [33]. Lower levels in females are mainly ascribed to the menstrual cycle. A study by Jahanshad et al. suggests that blood iron, as substantiated by transferrin levels, may impact WM fibre integrity, possibly due to elevated WM iron concentrations [34]. This study points towards the possibility that body iron and brain iron stores are better correlated than formerly thought [35]. Although we found non-significantly higher R2* values in all non-MS males compared to all non-MS females, separate analysis by sex (Fig 4) still demonstrates differences between all female groups. This suggests that although sex may augment the differences due to the higher percentage of males in the siblings group, these sex differences do not solely explain the observed R2* elevation of siblings versus HC. We attribute the lack of significance between the male groups to the small number of male participants (see Table 1). The small number of age- and sex-matched twins in this study does not allow to analyze whether the findings for the sibling group were driven by the even stronger genetic predisposition for MS encountered in twins. A study in a much larger group and with follow up data is needed to test the possible predictive power of R2* increase for conversion to MS.

### Previous MR sibling studies

Differences in neuroimaging outcomes between siblings of patients with MS and unrelated controls were previously reported. Asymptomatic relatives of patients with MS had a higher volume of WM signal abnormalities compared to non-related controls [36]. Three subjects from each non-MS group in our study presented WM hyperintensities on T2 weighted images and were excluded from the analysis. This number agrees with a previous siblings study [36]. A study using magnetization transfer ratio in a large cohort, albeit at 1T, showed that focal brain abnormalities occur in first-degree relatives of MS, that are indistinguishable from those of MS [37]. Similarly, no significant difference in magnetization transfer was seen between 15 siblings and 12 unrelated controls for all WM [38].

### R2* reduction in MS patients and its relation to disability index

The overall reduction in R2* in the MS group, i.e. reduced c_0_, could be due to a reduction in iron and/or myelin or due to an increase in water content. In a recent pathological study on a control and MS brain samples, the non-lesional WM of MS brains presented a reduction in non-heme iron within oligodendrocytes and myelin, which was correlated with disease duration [39]. Such a reduction in iron content could explain the observed decrease in R2* in the MS group. On the other hand, the reduced R2* in non-lesional WM is also in agreement with reduced myelin content in these areas in patients with MS [40]. The NAWM of patients with MS exhibits pathological changes, such as axonal damage, myelin density reduction, inflammation, microglial activation and loss of axons [8]. Each of these changes affects the transverse relaxation rates R2 and R2*, because modifications of the imaging voxels composition result in alterations of the spin-spin relaxation. For instance, the reduction of magnetization transfer in NAWM preceding the appearance of lesions [24] and the reduction of myelin water fraction in NAWM by 16% detected via a decrease in the short component of the T2 relaxation times [25] are both indicative of tissue integrity loss beyond overt lesions.

Several histopathological studies showed that there is axonal damage in early disease stages [23, 41], while DAWM was shown to be a particular feature of progressive MS [8], when the formation of new active MS lesions has become rare. Good agreement between T1 and T2 values and DAWM was also reported [42, 43]. The model (Eq 1) fails to fit lesional tissue (data not shown), likely due to the low number of lesion voxels and due to the reduced directionality arising from demyelination and axonal loss. Therefore, we only report the average R2* values within all lesions, which is significantly smaller than R2* in WM and NAWM of all participants.

The reduction of NAWM R2* values was significantly more pronounced in MS cases with a higher progression index, indicating a relation of WM R2* reduction with disease severity. All abovementioned reasons, i.e. iron or myelin loss or increase in water content could lead to a reduction of R2*, or a combination of these factors might be responsible for this relation.

This study has some limitations. The MS cohort consists of 32 patients with RRMS, 4 with SPMS, and 3 with PPMS. While a separate analysis of the RRMS patients led to essentially the same result as in the entire cohort, the small number of progressive patients did not allow for the detection of any differences between the disease subtypes. The use of a rapid low resolution DTI scan may be seen as another limitation. However, due to the slow variation of R2* with fibre orientation, this scan is sufficient for the measurement of WM orientation.

The differences between the three groups are governed by an offset, which is defined by the model’s orientation independent term c_0_, rather than the isotropic and anisotropic terms c_1_ and c_2_, suggesting that non-orientation dependent mechanisms dominate the observed R2* differences in normal WM, NAWM and DAWM.

## Supporting Information

S1 File R2* curves. This file containes the R2* as a function of angle for each subject as well as all subject specific information, including group (control, sibling, patient), age, sex, EDSS and disease duration.
(ZIP)
